# BK polyomavirus genotype IV isolates from virus-specific T-cell therapy recipients

**DOI:** 10.1128/mra.00625-25

**Published:** 2025-08-27

**Authors:** Tiana A. Walder, Heidi L. Meeds, Michael S. Grimley, Steven B. Kleiboeker, Assem Ziady, Anthony Sabulski, Sonata Jodele, Alix E. Seif, Stella M. Davies, Benjamin L. Laskin, Jason T. Blackard, Jeremy D. Rubinstein

**Affiliations:** 1 Division of Gastroenterology and Hepatology, College of Medicine, University of Cincinnati 2514, Cincinnati, Ohio, USA; 2 Division of Bone Marrow Transplantation and Immune Deficiency, Cincinnati Children's Hospital Medical Center 42910, Cincinnati, Ohio, USA; 3 Eurofins Viracor Laboratories 205259 , Lenexa, Kansas, USA; 4 Department of Pediatrics, University of Cincinnati College of Medicine 12303, Cincinnati, Ohio, USA; 5 Perelman School of Medicine, University of Pennsylvania 6572, Philadelphia, Pennsylvania, USA; 6 Division of Oncology, Children’s Hospital of Philadelphia, Philadelphia, Pennsylvania, USA; 7 Division of Nephrology, Children’s Hospital of Philadelphia, Philadelphia, Pennsylvania, USA; 8 Division of Oncology, Cancer and Blood Diseases Institute, Cincinnati Children's Hospital Medical Center 549412, Cincinnati, Ohio, USA; Queens College Department of Biology, Queens, New York, USA

**Keywords:** BK polyomavirus, virus-specific T-cell therapy, transplantation, viral diversity, genotype

## Abstract

BK polyomavirus (BKPyV) can cause disease in transplant recipients. Virus-specific T-cell (VST) therapy is effective in decreasing virus levels in many individuals. BKPyV genetic variation can impact replication kinetics and immune responses. Here, we present the near full-length BKPyV genome sequences from two VST recipients in which genotype IV was isolated.

## ANNOUNCEMENT

A member of the *Polyomaviridae* family and *Betapolyomavirus* genus, BK polyomavirus (BKPyV) is one of 14 identified human polyomaviruses (1). Primary infection typically occurs in childhood and is asymptomatic. Infection is life-long and persists in an inactive state in the epithelium of the kidneys, ureters, and bladder (2, 3). Transplantation and immunosuppression can lead to viral reactivation and BKPyV-associated diseases, including BKPyV-associated nephropathy (BKVAN), hemorrhagic cystitis, and thrombotic microangiopathy (4–6). There is no FDA-approved vaccine for BKPyV. However, recently, virus-specific T-cell (VST) therapy has emerged as a promising treatment for the reduction of BKPyV viral loads and disease-associated symptoms (7–9). Viral diversity—specifically within the large T antigen—can impact immune response (10). Hence, a comprehensive understanding of the viral diversity present within this unique treatment population is essential.

Near full-length BKPyV genotype analysis was performed on two VST recipients residing in the United States. Subject 1 is a 73-year-old male with a history of end-stage renal disease who received a renal transplant. Subject 2 is a 75-year-old male with a history of stem cell transplantation. He developed atypical hemolytic uremia syndrome following transplantation leading to stage 5 chronic kidney disease for which he received a renal transplant. Both subjects developed BKPyV viremia and BKVAN. At the time of infusion, BK viremia was 163,000 and 1,087,217 IU/mL, respectively, which decreased to 48,900 and 571,249 IU/mL after 1 month.

Urine samples taken at the time of infusion were processed according to published methods (11). Briefly, viral DNA was extracted from 1 mL of urine utilizing the QIAGEN QIAamp UltraSens Virus Kit. Rolling circle amplification was performed with the Cytiva Life Sciences TempliPhi kit followed by linearization utilizing the BamHI restriction enzyme. Full-length PCR was performed using the QIAGEN UltraRun LongRange PCR Master Mix with the primers BK1731F (5′ GGGGGATCCAAGATGAAAACCTTAGGGGCTTTAG 3′) and BK1731R (5′ GGATCCCCCATTTCTGGGTTTAGGAAGCATTCTAC 3′), and the product was run on a 1% agarose gel and extracted utilizing the QIAquick Gel Extraction Kit (12).

Next-generation sequencing (NGS) was performed on PCR products. Library preparation was completed using the NEBNext Ultra II FS DNA Library Prep Kit and sequenced on an Illumina NextSeq 2000 with the setting paired-end 2 × 61 bp. Quality control was analyzed using FastQC, and no reads were flagged as poor quality. Consensus sequences were generated by mapping reads to the reference genome AB263926 (genotype Ia) using UGENE 52.0 and the Bowtie2 method. Data regarding the raw and assembled reads are summarized in Table 1 (13). All tools were run with default parameters.

**TABLE 1:** Characteristics of NGS raw and assembled reads

	Raw reads			Consensus sequence	
Subject	Number of reads	Coverage (%)	Average read depth (×)	Length (bp)	GC content (%)
1	1,723,159	100	16,805	5,141	39
2	1,841,078	100	16,997	5,141	39

**Fig 1:**
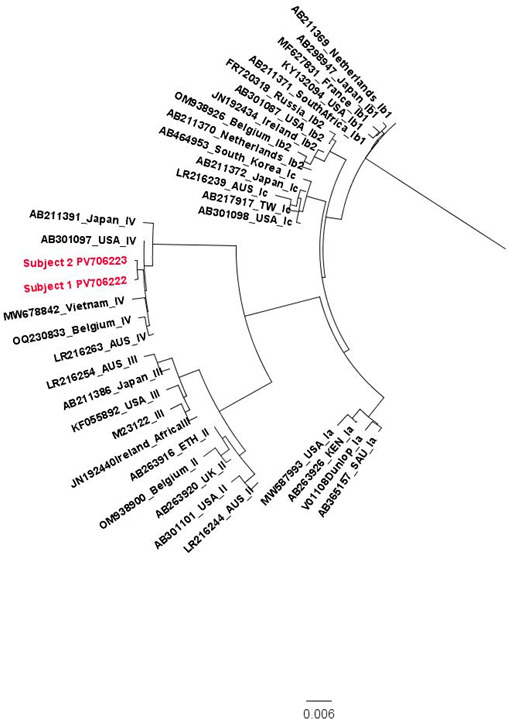
A multiple sequence alignment was carried out utilizing ClustalX 2.1 with pairwise alignment parameters. A phylogenetic tree was generated using the neighbor-joining algorithm. Five reference sequences were selected to represent each major BKPyV genotype (Ia, Ib1, Ib2, Ic, II, III, and IV), such that each reference represented a different country of origin. References are labeled with their GenBank accession number, country of origin, and BKPyV genotype. The subject sequences are highlighted in red and are labeled with their GenBank accession number.

A multiple sequence alignment was performed in ClustalX 2.1 with the consensus sequences along with references of known BKPyV genotype extracted from GenBank (14). An unrooted phylogenetic tree was generated and visualized in FigTree 1.4.4 (15). Both subject sequences clustered with genotype IV references (Fig. 1). Pairwise genetic distances were calculated in Mega 11.0 using the Kimura 2-parameter model (16). Subjects 1 and 2 exhibited 99.85% and 99.75% nucleotide similarity, respectively, with their closest related reference sequence (AB301097) from the United States.

## Data Availability

Raw sequence data are available under BioProject PRJNA1198305 under accession numbers SRX28935873 and SRX28935874. The consensus BKPyV genome sequences are available un der GenBank accession numbers PV706222 and PV706223.
